# Tracking androgens in female elite athletes: menstrual cycle and hormonal contraceptive effects

**DOI:** 10.1186/s12905-026-04344-y

**Published:** 2026-03-13

**Authors:** Jana Nolte, Sven C. Voss, Annekathrin Martina Keiler, Emily Büthe, Kirsten Legerlotz, Petra Platen

**Affiliations:** 1 Department of Sports Medicine and Sports Nutrition, Faculty of Sport Science, Ruhr-University Bochum, Bochum, Germany; 2 Institute of Doping Analysis and Sports Biochemistry Dresden (IDAS), Kreischa, Germany; 3 Environmental Monitoring & Endocrinology, Faculty of Biology, TU Dresden, Dresden, Germany; 4 Department of Movement and Training Sciences, Institute of Sport Science, University of Wuppertal, Wuppertal, Germany

**Keywords:** Testosterone, Pill, Hormones, Cycle-based training, Endocrinology

## Abstract

**Background:**

Understanding variations in the androgen profile is crucial for interpreting hormone data and developing cycle-based training strategies tailored to individual athlete needs. Therefore, this study investigates the androgen concentrations of elite female track and field athletes across various hormonal conditions, including natural eumenorrheic cycles, cycles under hormonal contraception, and cycles with menstrual disturbances.

**Methods:**

Using a prospective, longitudinal observational cohort design, 22 athletes (15 naturally cycling, 7 using hormonal contraception) were monitored for up to three consecutive cycles. The study utilised daily or near-daily urine sampling to analyse urinary steroid hormones (including testosterone and its metabolites) via gas chromatography-coupled mass spectrometry (GC-MS/MS).

**Results:**

Results demonstrated that in athletes with ovulatory menstrual cycles, urinary androgen concentrations, particularly testosterone, varied significantly by phase, peaking around the late follicular phase (lateFP: 7.35 ± 6.25 ng/mL). In contrast, athletes using combined oral contraceptives exhibited significantly suppressed androgen concentrations throughout the cycle compared to the natural cycle group, with mean urinary testosterone levels being notably lower (2.55 ± 2.91 ng/mL vs. 5.45 ± 4.31 ng/mL; *p*<0.001). These pill users also showed elevated concentrations of the inactive 5β-reductase metabolites (5β-androstanedione and 5β-androstanediol). Cycles with menstrual disturbances, such as luteal phase deficiency, also displayed altered and often lower concentrations of all urinary androgens.

**Conclusion:**

These findings highlight the significant influence of both endogenous menstrual cycle dynamics and exogenous hormone interventions on androgen availability, underscoring the necessity of considering the individual hormone status for performance optimization and athlete health management.

**Supplementary Information:**

The online version contains supplementary material available at 10.1186/s12905-026-04344-y.

## Key points

• Androgen suppression by contraception: Androgen concentrations, particularly urinary testosterone, are significantly suppressed and remain consistently low throughout the cycle in elite female athletes using combined oral contraceptives compared to naturally cycling athletes.

• Cyclical fluctuation in natural cycles: During the natural, ovulatory menstrual cycle, urinary androgen concentrations show systematic fluctuations, with testosterone and its metabolites peaking in the late follicular phase, immediately prior to ovulation.

• Need for individualised interpretation: Both menstrual disturbances and hormonal contraceptive use fundamentally change the androgen profile, emphasising that understanding individual androgen availability and considering contraceptive type and dosage is essential for performance strategies and accurate interpretation of hormonal data in elite female athletes.

**Supplementary Information:**

The online version contains supplementary material available at 10.1186/s12905-026-04344-y.

## Background

The role of anabolic steroid hormones in athletic performance has been extensively studied. The probably best studied anabolic steroid hormone is testosterone, which is positively correlated with specific performance metrics, including explosive power and lean muscle mass, which are vital for success in highly competitive sports like athletics [1, 2]. Furthermore, it is a key modulator of motivation and exercise performance [3]. However, studies have been predominantly performed with males only and research in female athletes remains limited as standardised research approaches are more challenging due to the complexity introduced by natural hormonal fluctuations across the menstrual cycle and the influence of hormonal contraceptives [4, 5]. This is unfortunate, as androgen hormones significantly contribute to the observed performance differences between sexes [6], highlighting the complexities surrounding female athletes with male hormone profiles, such as transgender women [7]. As the field continues to grapple with the effects of androgen manipulation, understanding natural variations in androgen levels can improve the interpretation of hormone data and help distinguish endogenous from exogenous androgen influence [8–10]. As a result, the measurement and interpretation of sex hormones is particularly important in the female athlete population for the further understanding female androgen profiles and furthermore to develop cycle-based training strategies tailored to the individual needs of athletes [11–13]. Understanding the androgen profile, including testosterone and its precursors such as androstenedione, is crucial for comprehending how these hormones may influence performance outcomes and training adaptation in female athletes [3, 14].

Because these effects ultimately depend on how androgens are generated and regulated, it is useful to consider their biosynthetic origins. The Δ5 (D5) steroidogenic pathway refers to the biosynthesis of steroid hormones from cholesterol and pregnenolone, and plays a critical role in the production of various steroid hormones, including androgens, estrogens, and progestogens produced by both the adult adrenal glands and gonads [15–17]. Fluctuations in estrogen and progesterone levels throughout the menstrual cycle reflect alterations in the activity of steroidogenic enzymes involved in this pathway, which in turn influence androgen production [18, 19]. As early as in the 1970s and 1980s, research highlighted fluctuations in testosterone and androstenedione levels across the menstrual cycle [20–22] observing a gradual increase in androgen concentrations during the follicular phase, with testosterone levels peaking around mid-cycle followed by a decline during the luteal phase. More recent studies have confirmed these fluctuations, often emphasizing a pronounced testosterone peak around ovulation [23–25]. These studies did not include the direct context of competitive and elite sports, nor did they feature such a close-meshed sampling with reliable menstrual cycle phase determination, that cycle-dependent testosterone curve can be recognized in the context of significant cycle fluctuations.

Regarding hormonal contraception, the use of combined pills containing estrogen and progestin compounds can significantly influence circulating androgen levels, potentially affecting performance and physiological outcomes in female athletes [18, 26–28]. However, here too, the available studies are insufficient to make clear recommendations on the use and impact of oral contraceptives in competitive and elite sports.

In addition, menstrual dysfunctions, which can occur along a spectrum from subclinical disorders, including luteal phase deficiency and anovulation, to clinical disorders, including oligomenorrhea and amenorrhea, are common in physically active women and may also impact androgen levels [12]. However, it remains unclear how specific forms of menstrual dysfunction quantitatively modify androgen availability and whether these alterations translate into meaningful differences in athletic populations.

Addressing these research gaps on androgen availability across verified natural menstrual cycles and hormonal contraception is essential, as misconceptions about cycle dynamics can compromise performance optimization and athlete health while a clearer picture of healthy cycles, dysfunctions, and contraceptive effects will refine our understanding of female physiology and interactions with adaptive processes [11, 29].

As the hormonal profile in different phases of menstrual cycle, with menstrual cycle disturbances and with the use of hormonal contraceptives may influence athletic performance and strength training adaptations [30], there is a growing need for a deeper understanding how anabolic sex steroid hormones are secreted and metabolised in those different hormonal conditions.

In this study, we aim to provide insights into the androgen profiles of elite female track and field athletes across normal and disturbed menstrual cycles and with hormonal contraceptive use.

We hypothesize that the androgen profile is suppressed both in response to exogenous hormonal intake and in the presence of menstrual cycle disturbances, while systematic fluctuations in androgen levels are expected to occur during the natural menstrual cycle, particularly immediately prior to ovulation.

## Methods

### Study design

This study follows a prospective, longitudinal observational cohort design without any experimental manipulation or intervention affecting the athletes’ behavior. The study period covered three consecutive menstrual cycles or a maximum of 90 days between June 1st and December 31st, 2022, during which the female track and field athletes systematically monitored their menstrual cycle, including performance-relevant biophysical, psychological, and psychosocial parameters. The current study was part of the DLV-Cycle-Tracking project (10.17605/OSF.IO/XBK7C). The present analysis focuses exclusively on the evaluation of biophysical parameters used to determine the menstrual cycle phases and to assess the individual androgen profile.

The study was positively evaluated by the independent ethics committee of the Faculty of Sport Science at the Ruhr University Bochum and performed in accordance with the Declaration of Helsinki (EKS V 06/2022). All participants received detailed information about the study procedures and data usage, both during several information sessions and in written form. Written informed consent to participate was obtained from all participants, or from their legal guardians in cases where participants were under 18 years of age.

### Participants

22 elite female track and field athletes (24 ± 5,7 y; 174 ± 6,1 cm; 69 ± 10,4 kg) from various disciplines (running (*n* = 8), jumping (*n* = 7), throwing (*n* = 7)) participated in this longitudinal study. Participants were classified as highly trained (Tier 3–5, athletes successfully competing at national or international events) [31]. Fifteen participants reported having a natural menstrual cycle, including one using a copper intrauterine device (IUD), while 7 participants used hormonal contraception such as combined oral contraceptives (*n* = 5) and IUDs (*n* = 2) (Table 1).

**Table 1:** Cohort description and comprehensive information on hormonal contraceptive use

Number	Contraceptive method	Details
14	None	
1	Copper IUD	
5	Oral contraceptive	Asumate 30 (0.15 mg levonorgestrel, 0.03 mg ethinylestradiol);
		Desofemine (0.15 mg desogestrel, 0.03 mg ethinylestradiol);
		Evaluna 20 (0.1 mg levonorgestrel, 0.02 mg ethinylestradiol);
		Evaluna 30 (0.15 mg levonorgestrel, 0.03 mg ethinylestradiol);
		Microgynon (0.15 mg levonorgestrel, 0.03 mg ethinylestradiol)
2	IUD	Mirena (52 mg levonorgestrel);
		Jaydess (13.5 mg levonorgestrel)

### Measurements

Data collection was conducted both by the athletes themselves and by trained staff members from the Department of Sports Medicine and Sports Nutrition at the Faculty of Sport Science, Ruhr University Bochum, and/or staff at the Olympic Training Centers (OSPs).

Data were collected via three main approaches: Self-report and self-sampling via the AthleteMonitoring app (https://www.athletemonitoring.com/), daily logs, and home-based sampling routines; supervised data collection at the athlete’s residence or training site (including OSPs); and remote self-monitoring using specific wearable technology.

**Fig. 1:**
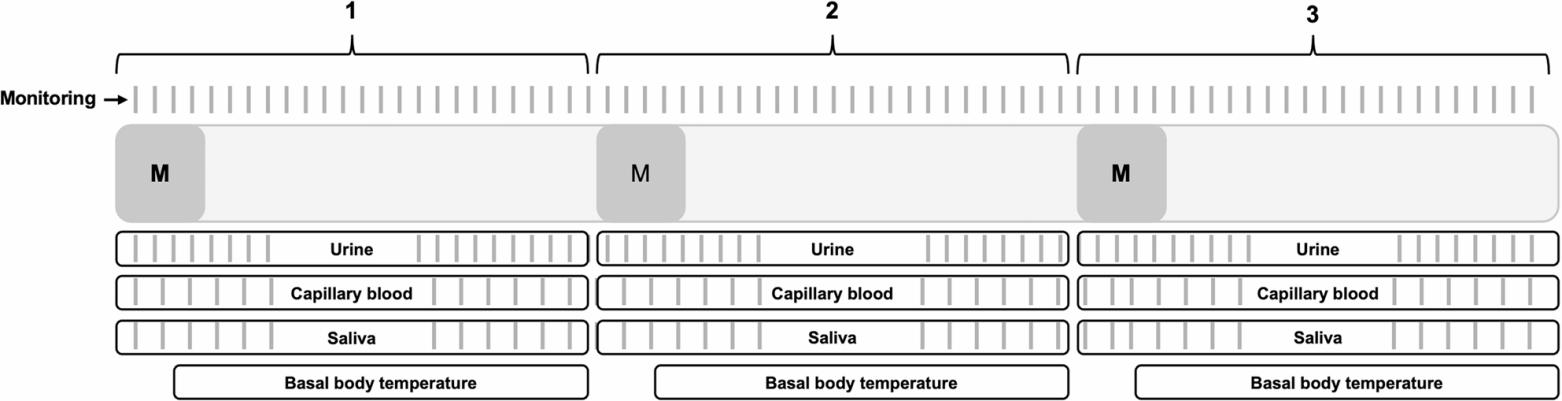
Study design and measurement schedule over three consecutive menstrual cycles. Monitoring included specific monitoring questions about onset und duration of bleeding on a daily basis. Additionally, basal body temperature (via OvulaRing), urine collection was performed daily, saliva sampling and capillary blood sampling were performed on specific days. “M” indicates the onset of menstruation in each cycle. All parameters were recorded longitudinally across the three-cycle observation period

Cycle monitoring was conducted continuously over three consecutive menstrual cycles, with a focus on biophysical parameters used to determine individual menstrual cycle phases (adapted three-step [32, 33] menstrual mapping) and to assess hormonal profiles (see Fig. 1).

A calendar-based approach was used to determine cycle length by documenting the onset of menstrual bleeding (step 1). Ovulation was determined based on the core body temperature profiles (step 2) recorded by a temperature sensor designed for intravaginal use (OvulaRing^®^, VivoSensMedical GmbH, Leipzig, Germany). The sensor recorded core body temperature every five minutes - resulting in 288 data points per day - with particular emphasis on nocturnal values. It was worn daily to generate 24-hour temperature profiles that support the identification of cycle phases. For this purpose, the lowest nightly temperature values were analysed, as they most accurately reflect basal resting temperature. The detection of ovulation followed the “three-over-six” rule, whereby an ovulatory shift was confirmed if at least three consecutive higher temperature readings exceeded the highest of the preceding six lower values by at least 0.2 °C [34]. This method allows for retrospective identification of ovulation with high specificity, based on sustained thermogenic changes triggered by the luteinizing hormone surge. In participants (or cycle) who did not use the temperature sensor (e.g. participants under the age of 18), ovulation was approximated based on secondary indicators, most notably in rising progesterone levels.

**Fig. 2:**
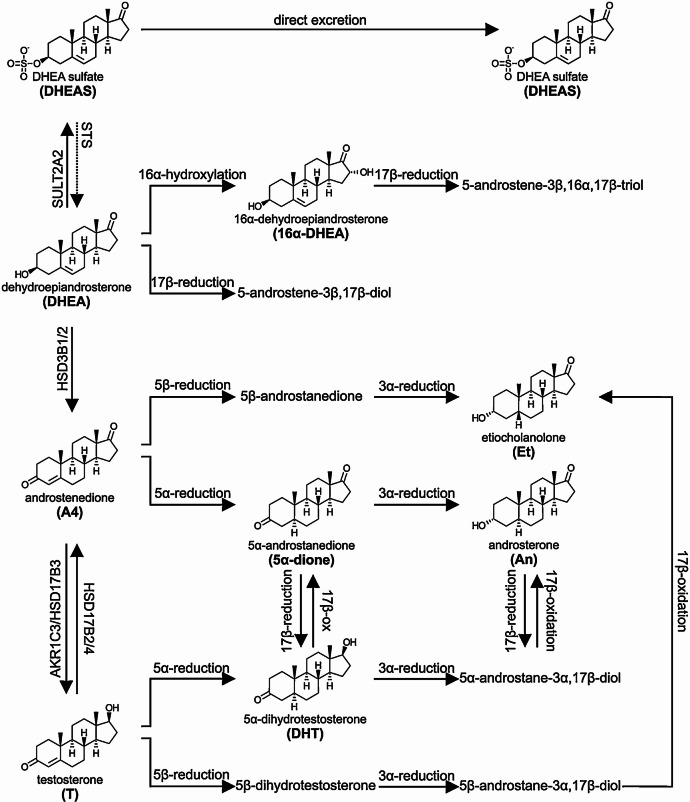
Schematic representation of androgen precursor metabolism to major urinary metabolites. Key serum androgens and precursors are shown on the left, with their major urinary metabolites on the right. The formation of 5α-dihydrotestosterone (DHT) from testosterone is reflected only by urinary androsterone (Figure from Schiffer et al. [15] [CC BY 4.0 license])

Capillary blood samples (250 µl, EDTA microtainer) were obtained before training on Mondays, Wednesdays, and Fridays, and were analysed for plasma progesterone by liquid chromatography with mass spectrometry coupling (LC-MS/MS) [15, 35]. In parallel, morning saliva samples were collected each Monday, Wednesday, Friday and Sunday as an additional and alternative measuring method for progesterone. The samples were analysed by ELISA technique (IBl-international, Tecan Trading AG, Switzerland). Plasma and saliva progesterone were collected to evaluate luteal function (step 3). The urine samples, taken as first morning urine, were collected daily by the athletes themselves, stored frozen and analysed in batches after the survey period for each day or every other day. Urinary steroid hormones (testosterone, 5α-androstanedione, 5β-androstanedione, 5α-androstanediol, 5β-androstanediol, androsterone, etiocholanolone) were analysed by gas chromatography-coupled mass spectrometry (GC-MS/MS) [36]. Specific gravity correction was applied for calculation of urinary steroid concentrations. The synthetic pathways are shown in Fig. 2. The detailed analytical procedure is provided in supplementary material A1.

All biological samples were stored under appropriate conditions, either refrigerated or frozen, and were shipped approximately once per cycle to the Department of Sports Medicine and Sports Nutrition at Ruhr University Bochum for further processing. Urine and blood plasma analyses were performed in several batches by the Institute of Doping Analysis and Sports Biochemistry (IDAS) in Kreischa, Germany.

### Menstrual cycle analysis and phase classification

All menstrual cycles were retrospectively classified as either eumenorrheic (cycle length between 21-35 days, ovulatory, luteal phase length above 10 days, progesterone rise in luteal phase) or dysfunctional. Menstrual dysfunctions (adapted from [37]) were defined as (a) oligomenorrhea, that is, menstrual cycle length greater than 35 d but less than 90 d [5]; (b) anovulation, that is, no ovulation detected by the basal body temperature measurement using the “three-over-six” rule [33, 34, 38]; (c) short luteal phase, that is, luteal phase shorter than 10 d [39]; (d) luteal phase deficiency, that is, progesterone concentration < 5 ng/ml in the luteal phase [33] or progesterone concentration in the luteal phase exceeded the individual menstrual phase baseline (in pg/mL) by a multiple of 1.85 [40]; and (e) missing data divided in no basal body temperature measurements (noT) and no progesterone measurements (noP).

**Fig. 3:**
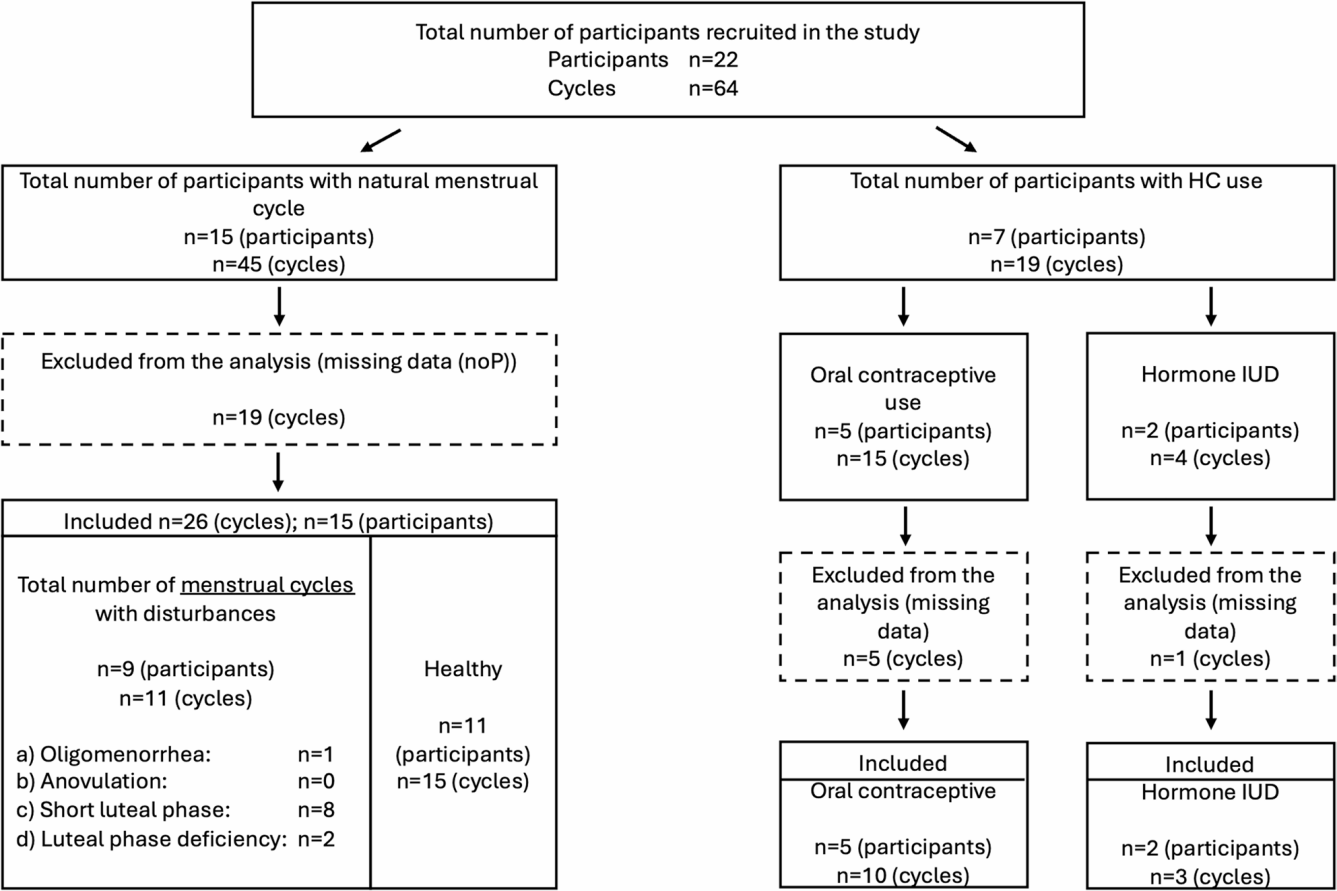
Flowchart of cycle inclusion

Of the 64 recorded cycles, 39 cycles were included in the final analysis (see Fig. 3).

Based on recommendations of Elliott-Sale et al. [11], the menstrual cycles were divided into seven phases as follows: earlyFP (day 1 to day 4 of the menstrual cycle), midFP (day 5 until lateFP), lateFP (two days before ovulation phase), OP (2 days before first temperature rise (ovulation day); expecting within ± 36 h of a luteinizing hormone (LH) surge), earlyLP (day 1 to day 5 of the luteal phase), midLP (6 to 8 days after ovulation day; expecting LH surge one day before), lateLP (day 9 of the luteal phase until onset of menstrual bleeding).

Oral contraceptive phases were determined as follows: pill-free (day 1 to day 7 without pill taking) and pill-taking phase (day 8 to day 28 while taking one pill every day).

Depending on the detection of ovulation, hormone profiles under hormonal IUD are presented either with allocation of menstrual cycle phases or described as anovulatory cycles.

### Statistics

The hormone levels were displayed graphically (without outliers) to visualize the differences between the individual phases of the menstrual cycle, oral contraceptive and IUD and to highlight hormonal fluctuations.

Raw concentrations were log-transformed to reduce skewness. Subsequently, values were z-standardized within each participant by subtracting the participant’s mean and dividing by the participant’s standard deviation. This within-participant standardization reduces between-participant baseline differences and facilitates phase-wise comparisons of relative changes. Statistical analyses were conducted for all participants with ovulatory cycles (groups: healthy menstrual cycle (MC), oligomenorrhea (A), short luteal phase (C), luteal phase deficiency (D)) as well as for users of combined oral contraceptives. To examine differences in hormone concentrations across menstrual-cycle phases and pill phases, linear mixed-effects models were fitted. These models included random intercepts to account for interindividual variability and the repeated-measures structure of the data. Fixed effects consisted of the menstrual-cycle phases (or pill phases), whereby each phase was contrasted exclusively against the late follicular phase in accordance with the hypothesis-driven testing strategy. *P*-values were adjusted using Holm adjustment.

The average of testosterone concentrations of all athletes (per group) was used for statistical comparison between eumenorrheic menstrual cycle and combined contraceptive pill use using the Mann-Whitney-U test (not normally distributed) to investigate whether there are differences in the hormone levels between the groups.

*P* values ≤ 0.05 will be considered to reflect statistical significance. Values will be reported as mean (95% confidence interval) unless indicated otherwise.

## Results

To examine phase-specific differences, we analysed ovulatory cycles (*n* = 26) and combined oral contraceptive (pill) cycles (*n* = 10). For group-specific comparisons, cycles were categorised as natural eumenorrheic (*n* = 15), menstrual disturbances (*n* = 11), oral contraceptive use (*n* = 10), and hormonal IUD use (*n* = 3). Menstrual disturbances comprised oligomenorrhea (*n* = 1), short luteal phase (*n* = 8), and luteal phase deficiency (*n* = 2).

**Fig. 4:**
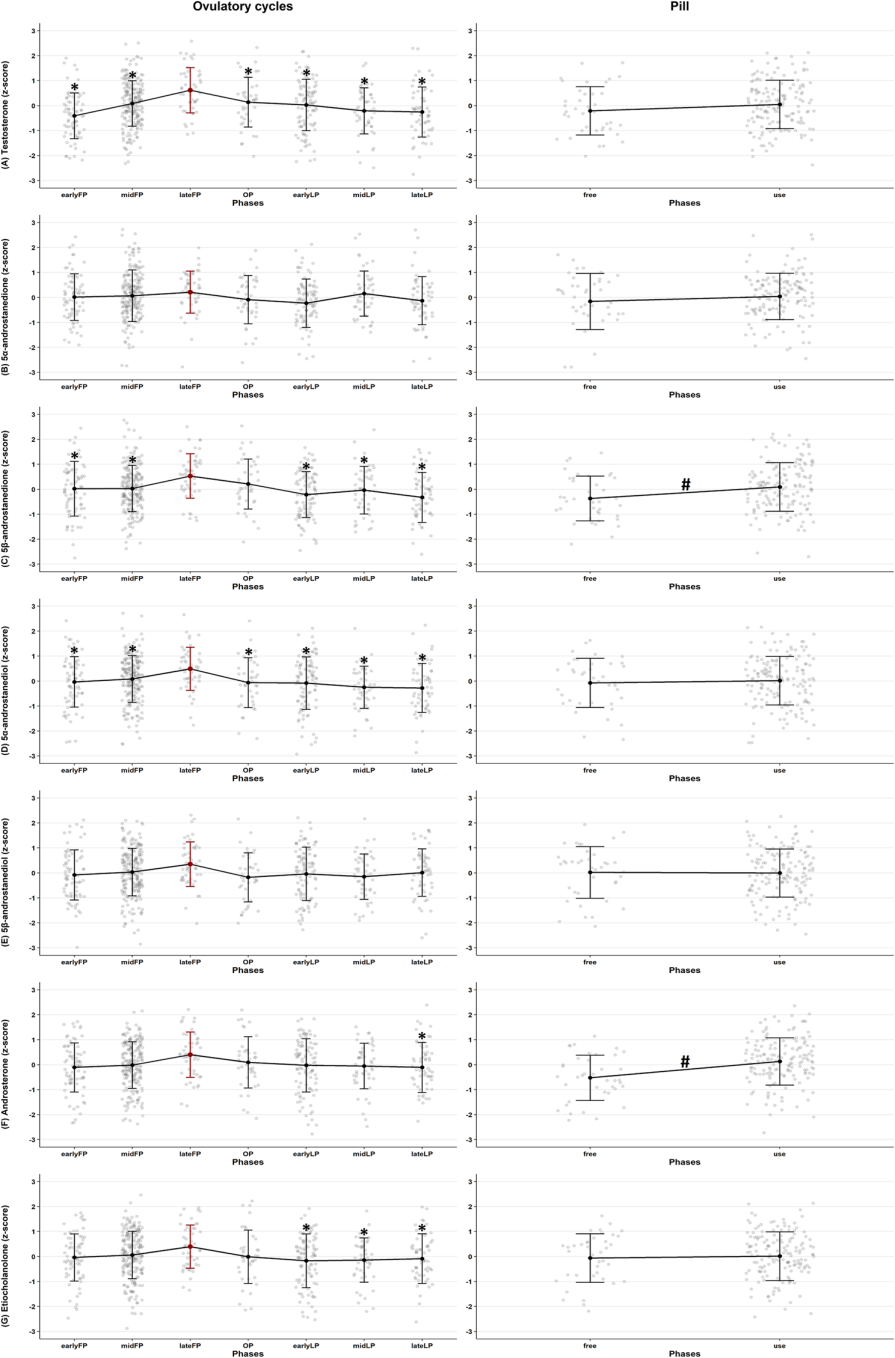
Urinary androgen profiles across phases of ovulatory menstrual cycles and combined oral contraceptive use. The figure shows log-transformed and z-standardised values for (**A**) testosterone, (**B**) 5α-androstanedione, (**C**) 5β-androstanedione, (**D**) 5α-androstanediol, (**E**) 5β-androstanediol, (**F**) androsterone, and (**G**) etiocholanolone. Left panel: individuals experiencing ovulatory menstrual cycles (group: MC, A, C, D). Right panel: individuals using combined oral contraceptives (pill). Hormone values are visualised across cycle or pill phases using line plots and scatter clouds. *Statistically significant phase differences within each hormone in ovulatory cycles, tested against the early follicular phase (red lines) using a linear mixed model. ^#^Statistically significant phase differences within each hormone in pill users, tested between free and use phases using a linear mixed model

In athletes with ovulatory menstrual cycles without any hormonal contraceptive use (*n* = 26), urinary androgen concentrations varied by phase for testosterone, 5β-androstanedione, 5α-androstanediol, androsterone, and etiocholanolone (Fig. 4). Testosterone and 5α-androstanediol were significantly different in the lateFP compared with all other phases (*p* ≤ 0.05). 5β-Androstanedione showed a similar pattern, with no difference between the lateFP and OP. For androsterone and etiocholanolone, the late follicular phase differed significantly from the luteal phase (both *p* ≤ 0.05). Among athletes using combined oral contraceptives, 5β-androstanedione and androsterone levels were significantly lower in the pill-free compared to the pill-use phases (*p* ≤ 0.05) (Fig. 4).

**Fig. 5:**
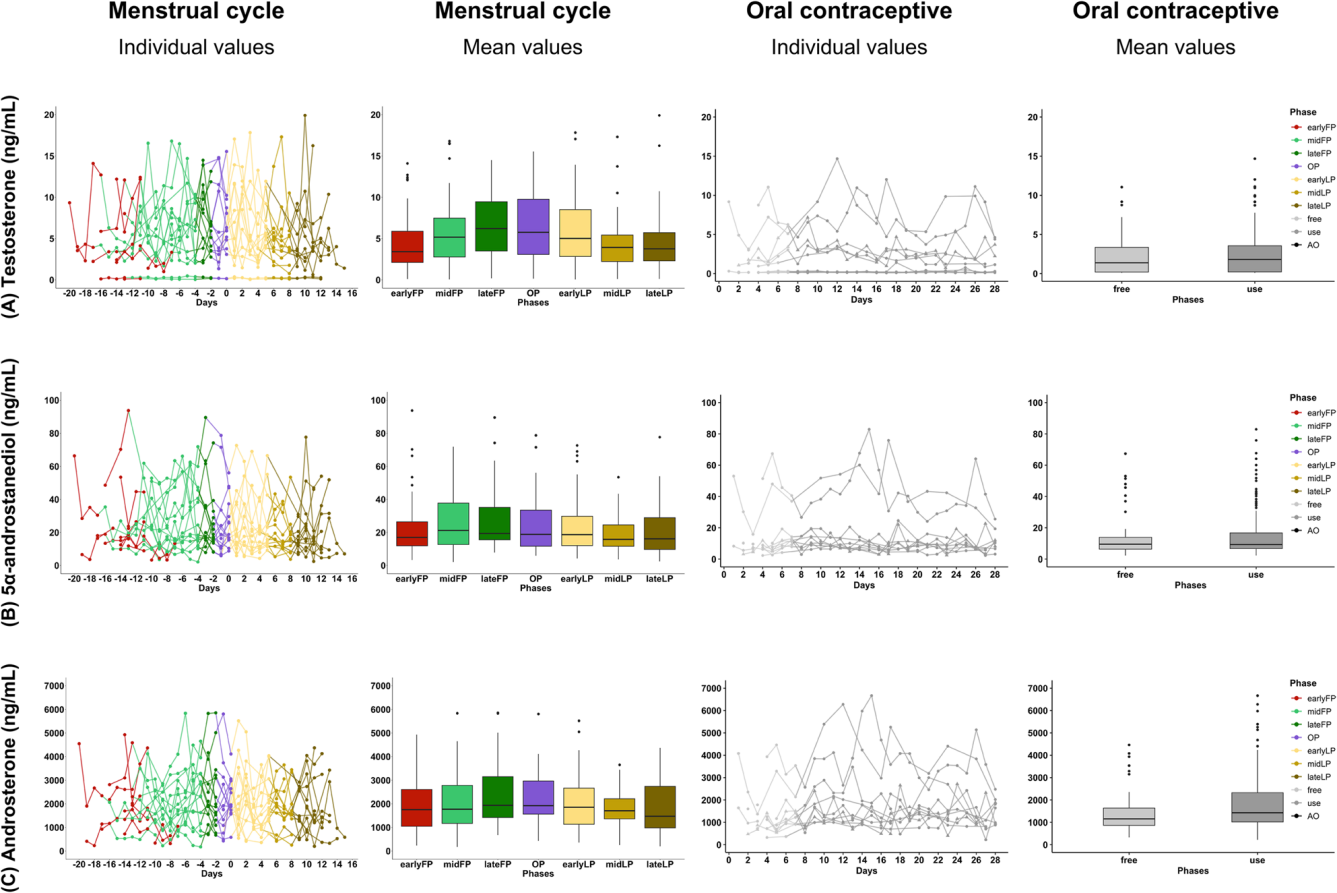
Urinary androgen profile of menstrual cycle and oral contraceptive. The hormones presented include **A**: testosterone; **B**: 5α-androstanediol; and **C**: androsterone. Data is shown for two groups: individuals undergoing a natural menstrual cycle (colored lines and colored box plots, left) (n_data_ = 332; n_cycle_ = 15) and individuals using combined oral contraceptives (grey lines and grey box plots, right) (n_data_ = 210; n_cycle_ = 10) (see supplementary material A3). Hormone levels are plotted as line graphs across days normalised to the estimated day of ovulation (day: 0; FP < -1; LP > 1) and summarised as box plots across menstrual or pill phases. Axes are scaled per hormone to improve visualization of temporal patterns. Outliers were deleted for visualization purposes. A lower-dose oral contraceptive (containing 0.1 mg levonorgestrel and 0.02 mg ethinylestradiol) is marked with triangles in the line plots

**Fig. 6:**
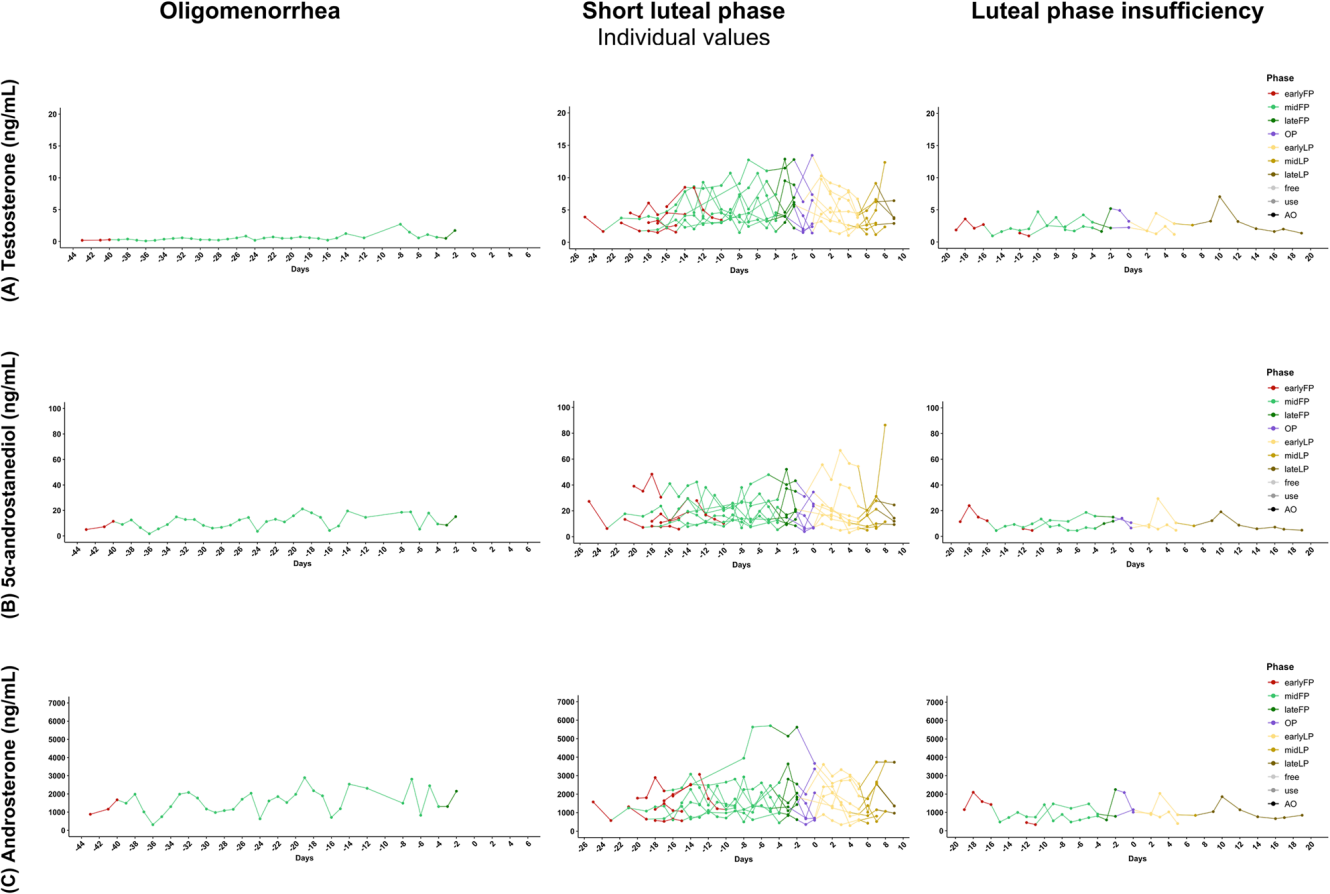
Urinary androgen profiles across menstrual cycle disturbances. Data is grouped by cycle type: oligomenorrhea (left) (n_data_ = 37; n_cycle_ = 1), short luteal phase (middle) (n_data_ = 169; n_cycle_ = 8), and luteal phase insufficiency (right) (n_data_ = 43; n_cycle_ = 2). The hormones presented include **A**: testosterone; **B**: 5α-androstanediol; and **C**: androsterone. Hormone concentrations are shown as line plots across days normalised to the estimated day of ovulation (day 0; FP < -1; LP > 1). Menstrual phases are color-coded in the plots. Axes are scaled per hormone to improve visualization of temporal patterns. Outliers were deleted for visualization purposes

**Fig. 7:**
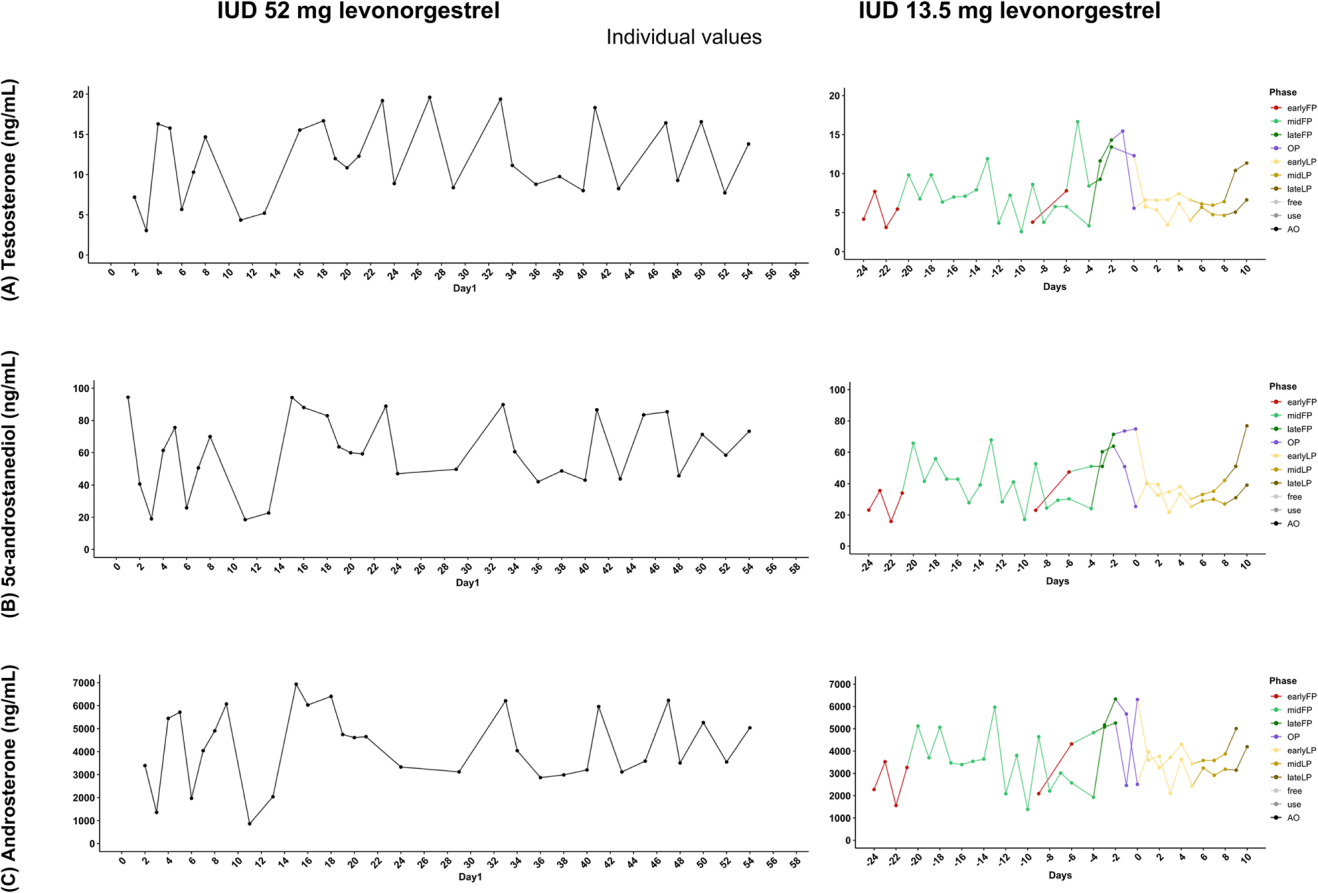
Urinary androgen profiles in individuals using levonorgestrel-releasing intrauterine devices (IUDs). The hormones presented include **A**: testosterone; **B**: 5α-androstanediol; and **C**: androsterone. Line plots show hormone concentrations over time in users of either a high-dose IUD (52 mg levonorgestrel; left) (n_data_ = 42; n_cycle_ = 1) or a lower-dose IUD (13.5 mg levonorgestrel; right) (n_data_ = 52; n_cycle_ = 2). For the lower-dose IUD group, days are aligned to the estimated day of ovulation (day 0; FP < -1; LP > 1). In cases where ovulation did not occur, days are shown in chronological order. Axes are scaled per hormone to improve visualization of temporal patterns. Outliers were deleted for visualization purposes

The absolute hormone concentrations for testosterone, 5α-androstanediol, and androsterone for each group are presented in Figs. 5, 6 and 7 (all hormones are presented in the supplementary material A3, A4, A5), shown as raw values and temporal trajectories as well as corresponding box plots for eumenorrheic menstrual cycle and pill users. Inferential phase-wise testing was only conducted on log-transformed, within-participant z-standardized values and is presented in Fig. 4.

In athletes with eumenorrheic menstrual cycles, mean testosterone concentrations were lowest in the early follicular phase (earlyFP: 4.64 ± 4.05 ng/mL), peaked before ovulation (lateFP: 7,35 ± 6,25 ng/mL) and declined again in the late luteal phase (lateLP: 4.55 ± 3.90 ng/mL) (see supplementary material A2). Similar patterns were seen in urinary androgen precursors and metabolites. Hormone profiles displayed considerable inter-individual variability in baseline levels, with each curve often remaining within its own hormonal range and not reaching the peaks or troughs seen in others. Notably, two testosterone curves in the naturally cycling group showed markedly suppressed testosterone excretion, despite following an apparently natural menstrual pattern.

Among pill users, testosterone (*p* ≤ 0.001), 5α-androstanediol (*p* ≤ 0.001), and androsterone (*p* = 0.022). concentrations remained consistently lower throughout the entire pill cycle in comparison to naturally cycling athletes. This suppression was consistent across all individuals and time points. Mean urinary testosterone levels (2.55 ± 2.91 ng/mL) under pill use were significantly lower than those in the natural cycle group (5.45 ± 4.31 ng/mL, *p* ≤ 0.001). In contrast, concentrations of 5β-androstanedione (*p* ≤ 0.001) and 5β-androstanediol (*p* = 0.034) were significantly higher in pill users than in the natural cycle group. No significant differences were observed for 5α-androstanedione or etiocholanolone (*p* ≤ 0.05).

One participant used a lower-dosed combined oral contraceptive (0.1 mg levonorgestrel, 0.02 mg ethinylestradiol), which is indicated by triangles instead of dots in the line plots in Fig. 5. Despite the lower concentration, this case aligned centrally within the overall dataset.

Cycles with menstrual disturbances exhibited altered androgen profiles (Fig. 6). In the single case of oligomenorrhea, a prolonged follicular phase was observed, accompanied by a delayed but distinct increase in testosterone and its metabolites later in the cycle. No data points were available for ovulation, and the luteal phase was notably short, lasting only five days. In short luteal phase cycles, testosterone levels increased during the follicular and ovulatory phases but declined prematurely, consistent with shortened luteal function. Cycles with luteal phase deficiency exhibited very low concentrations of all urinary androgens, without any discernible pattern.

Androgen profiles among IUD users varied depending on the hormonal dose (Fig. 7). The athlete using the 52 mg levonorgestrel IUD did not ovulate during the 90-day observation period. Her urinary androgen profile over 60 days showed highly fluctuating androgen levels with considerable variability, but no observable cyclical pattern. In contrast, the 13.5 mg IUD was associated with ovulation and a biphasic cycle, along with more stable and structured hormone profiles. These included moderate mid-cycle elevations in testosterone and its metabolites, followed by a distinct and immediate decline.

## Discussion

To our knowledge, this study is the first to systematically investigate urinary steroid hormone profiles at a high frequency in competitive female athletes across distinct menstrual cycle conditions, including natural eumenorrheic cycles, cycles under hormonal contraception, and cycles with menstrual disturbances. In line with our hypothesis, androgen concentrations were significantly lower in athletes using exogenous hormonal contraception and appeared lower in athletes with menstrual cycle irregularities (not formally tested). In menstruating athletes with ovulation, we observed significantly higher androgen levels immediately prior to ovulation compared to other menstrual phases.

These findings underscore the importance of considering individual androgen profiles and their variability when interpreting hormonal data in athletic populations. Taken together, these urinary excretion patterns highlight systematic differences in androgen metabolism depending on the phase of the menstrual cycle and the use of hormonal contraceptives. Although urinary profiles do not quantify bioactive androgen exposure at the tissue level, they could help to generate hypotheses about periods of altered endocrine milieu for future studies to explore by linking urinary profiles with concurrent blood measures and performance or training response outcomes.

### Individual androgen profiles in athletic context

Individual androgen levels in female athletes are influenced by various factors, including genetic predispositions, health status, and the specific type and amount of training regimen the athlete undergoes [26, 41, 42]. While on average, our naturally menstruating cohort had urine testosterone concentrations of 5.45 ± 4.31 ng/mL it should be noted that androgen profiles varied a lot between individual athletes. While one athlete with an eumenorrheic cycle had an average testosterone concentration in urine of 0.14 ± 0.08 ng/mL, another athlete had an average concentration of 10.93 ± 3.35 ng/mL. Studies indicate that higher testosterone levels within the normal physiological range are associated with enhanced muscle mass, strength, and overall athletic performance among female athletes [6, 9, 43]. Furthermore, the occurrence of hyperandrogenism, which can be linked to conditions such as polycystic ovary syndrome, reflects a genetic predisposition influencing androgen levels and potentially yielding in performance advantages in competitive sports because of elevated testosterone levels [6, 44]. Genetic factors such as single nucleotide polymorphisms in androgen receptor genes may influence individual responses to androgens and training adaptations [45]. However, urinary excretion data cannot be used to classify hyperandrogenism or other clinical endocrine conditions without parallel serum and SHBG measurements. Although a comparison of mean values already provides initial insights into the cyclical nature of androgen release, it is more important to interpret inter-individual urinary androgen differences as variability in excretion/turnover.

### Menstrual cycle dynamics and exogenous hormonal influence

In both pathological and non-pathological conditions, physiological fluctuations in steroid hormone profiles across the menstrual cycle must be considered. Evidence suggests that testosterone levels may peak around ovulation, consistent with reports of substantial variability in androgen concentrations across different menstrual phases [8, 24, 46]. These variations are closely linked to the pulsatile secretion of LH, which regulates not only ovarian estrogen synthesis and secretion but also the phase-dependent release of ovarian androgens [23]. In relation to the menstrual cycle, estrogen and progesterone concentrations can fluctuate up to 15-fold across the menstrual cycle, interacting with testosterone in such a way that the effect of free testosterone on muscle mass often disappears when models are adjusted for estrogen or insulin [30]. For example, the cyclical expression of estrogen receptors ERα and ERβ in skeletal muscle indicates that the effects of testosterone may be indirectly modulated by menstrual phase–dependent receptor dynamics [30].

The reduction in androgen levels detected during oral hormonal contraceptive use reflects the intended pharmacological effect of inhibiting ovulation and maintaining a steady hormonal milieu [28]. Previous studies on daily urine profiles in women have shown that the use of oral contraceptives can suppress typical, cycle-related endocrine fluctuations and modify urinary steroid excretion patterns [47]. This is consistent with the more stable, and generally reduced, androgen excretion observed in the pill users of our study. As a result, it creates a stable yet overall diminished hormonal profile compared to the cyclical peaks and troughs characteristic of the natural menstrual cycle [28, 48]. The consistently reduced urinary androgen excretion observed in oral contraceptive users likely reflects a suppression of androgen synthesis. Given the roles of testosterone, androstenedione, and dehydroepiandrosterone in reproductive physiology, skeletal muscle development, and bone health [49], such reductions may have broader physiological implications. The elevated concentrations of the β-reductase metabolites (5β-androstanedione and 5β-androstanediol) in oral contraceptive users further support this hypothesis, as these metabolites are associated with reduced androgen receptor activity and represent metabolic inactivation pathways [15]. Such hormonal suppression could carry implications for athletic performance, as a consistently low androgen environment may constrain some of the physiological adaptations that are normally supported by higher and more variable androgen availability [1, 50].

In contrast to the urine testosterone fluctuations across the menstrual cycle, levels remain comparatively stable and lower during hormonal contraceptive use. This pattern aligns with previous findings suggesting that athletes using hormonal contraception may show reduced variation in strength performance throughout the cycle [51]. Such hormonal stability could offer potential advantages for athletes aiming for greater consistency in training and competition outcomes.

### New insights into androgen evaluation

Moreover, the distinction between steroid hormone concentrations in different biological matrices (i.e., saliva, blood, urine) is critical for understanding muscle adaptation. When attempting to interpret steroid hormones in saliva, it is important not only to use a valid immunoassay, but also to bear in mind that the interpretation of salivary steroid hormone levels remains a challenging task due to the large variability of the results compared to serum [52]. Serum and plasma concentrations reflect the acute hormonal state, while urinary excretion offers a more integrated and stable view of androgen metabolism over time [15, 42]. According to Schiffer et al. [15], urinary steroid profiles represent not only the production of hormones but also their subsequent metabolism and elimination, both of which may be influenced by physiological factors such as hormonal contraceptive use. As an example, emergency contraceptive intake has been shown to markedly alter urinary steroid patterns [53]. However, while testosterone and its metabolites can be detected in urine, these concentrations do not necessarily reflect the bioactive form that enters muscle cells.

While circulating androgens such as testosterone and androstenedione reflect the overall hormonal environment and are linked to muscle quality, serum and intramuscular testosterone levels do not correlate directly, and therefore blood fluctuations may not reflect local muscle hormone dynamics [30]. Intramuscular steroid concentrations appear to be maintained or even elevated via local steroidogenesis, particularly shown in postmenopausal women [54]. This locally produced pool of sex steroids supported by the presence of steroidogenic enzymes may have distinct, tissue-specific functions, potentially influencing intramuscular fat deposition and contributing to the regulation of neuromuscular characteristics independently of systemic hormone status [54]. Such variations highlight the necessity of considering both the absolute hormone levels and the sensitivity of androgen receptors, as adaptations in muscle response to training are influenced by both hormone availability and receptor density [55, 56].

Urinary steroid profiles generated through GC-MS/MS offer a detailed view of androgen metabolism, although their interpretation requires distinguishing between metabolites with biological activity and those that represent inactive by-products. Testosterone is the principal biologically active androgen. Metabolites such as 5α-androstanedione and 5α-androstanediol act as precursors and downstream intermediates within the 5α-reductase pathway and therefore reflect the biochemical capacity for the formation of 5α-dihydrotestosterone and stronger androgenic signaling [57, 58].

Metabolites produced through 5β-reduction, including 5β-androstanedione, 5β-androstanediol and etiocholanolone, lack androgen receptor activity and represent metabolic inactivation routes [15]. Androsterone, which constitutes the major 5α-reduced urinary end product, exhibits only minimal intrinsic androgenic potency but serves as a well-established marker of 5α-reductase activity in urine [59]. The relative proportion of 5α- and 5β-derived metabolites, for example the ratio of androsterone to etiocholanolone, is therefore frequently applied to evaluate androgen metabolism in research and applied settings [60]. Although the aggregated output of urinary androgen metabolites correlates with circulating testosterone and can reflect androgen exposure [61], it is important to note that urinary steroid measurements primarily represent systemic metabolism and excretion. Tissue-specific androgen action, in contrast, is determined by local intracrine processes and is not directly captured by urinary profiles [59].

Plasma analysis provides also detailed information on circulating androgens (e.g., testosterone, androstenedione, DHEA) and their binding to sex hormone-binding globulin (SHBG), which regulates bioavailability, making it well suited for detecting acute, diurnal, or menstrual phase–related fluctuations, but it is more sensitive to short-term influences such as stress, training, or hormonal contraceptive use [15]. Urine analysis, by measuring conjugated androgen metabolites such as androsterone and etiocholanolone that result from hepatic metabolism, offers an integrated and stable reflection of overall androgen turnover over extended periods, largely unaffected by short-term stress or exercise [15], although one study demonstrated that urinary steroid profiles are not entirely unaffected by physical exercise. This study showed that acute high-intensity training can cause transient elevations in several androgen metabolites, which may normalize within 24 h [42]. A combined plasma–urine approach therefore allows simultaneous assessment of immediate hormonal status, SHBG-related bioavailability, and long-term metabolic output, providing the most comprehensive picture of androgen profiles in female athletes.

### Limitations

Lastly, despite the methodological rigor of the study, including comprehensive hormonal assessments and continuous menstrual cycle monitoring, several limitations should be acknowledged. The moderate sample size may restrict the generalizability of the findings. For this reason, we decided to present the results in a mainly descriptive manner and only tested for differences when sufficient data was available (menstrual cycle vs. oral contraceptive). While the study was conducted in an uncontrolled, real-world training environment, offering valuable insights into the hormone profiles of elite athletes, this lack of control also introduces potential confounding factors related to training load, sleep, alcohol, illness, medication intake, dietary supplements, and environmental influences. Although training, sleep, alcohol consumption, and medication intake had to be documented in part, these factors (plus ethyl glucuronide values in urine), could not be specifically considered in this analysis and should be systematically addressed in future studies. Additionally, inter-individual differences in urinary testosterone excretion may partly reflect genetic variability in testosterone glucuronidation (e.g., UGT2B17 deletion), a factor that was not considered in the present study. Furthermore, urinary steroid profiles can be affected by sample stability and microbial activity. The accumulation of 5α- and 5β-androstanedione has been described as an indicator of urine degradation, highlighting the importance of careful sample handling and data screening [62].

Phase determination in the menstrual cycle did not rely on the gold-standard combination of repeated LH testing and serum progesterone measurements [5, 11, 63]. Instead, intravaginal temperature monitoring combined with plasma or salivary progesterone was employed [40, 64]. While these methods are more practical in the elite sports context and have shown high diagnostic value, they may not fully match the precision of gold-standard laboratory approaches. The classification of a healthy luteal phase length also remains challenging. Although luteal phases shorter than ten days are often considered insufficient [39], evidence suggests that some women with luteal phases of less than ten days still produce adequate progesterone levels and maintain normal fertility [65, 66].

### Practical applications

This study provides new insights into androgen fluctuations across different hormonal conditions, including the first detailed hormonal profiling of athletes using hormonal IUDs.

In summary, comprehending the influences on androgen levels, the cyclical nature of hormone fluctuations, the varying bioactive roles of these hormones across different matrices, and the complexities of receptor dynamics are essential for distinguishing physiological responses in elite female athletes. Understanding the cyclical nature of androgen hormone changes is an initial step that can help to optimize training by aligning workloads with periods of higher anabolic potential. These findings also highlight the importance of considering contraceptive type and dosage when interpreting hormone data, with implications for both performance strategies and sports regulations.

## Conclusion

This study provides concise evidence of androgen fluctuations across natural cycles, hormonal contraception, and menstrual disturbances, including the first detailed profiling of athletes using hormonal IUDs. Recognising cycle phase, contraceptive type/dose, and matrix-specific measures is essential for interpreting androgen data and optimising training, as pre-ovulatory peaks and OC-associated suppression shape hormone availability. These insights support aligning workloads with periods of greater anabolic potential and call for refined biomarker use and sport policy that account for female-specific endocrinology.

## Supplementary Information

Supplementary Material 1.

## Data Availability

The datasets used and/or analysed during the current study are available from the corresponding author on reasonable request.

## Abbreviations

A: Group: Oligomenorrhea
C: Group: Short luteal phase
D: Group: Luteal phase deficiency
D5: Δ5 steroidogenic pathway
DHEA: Dehydroepiandrosterone
DHT: 5α-dihydrotestosterone
EDTA: Ethylenediaminetetraacetic acid
ELISA: Enzyme-linked immunosorbent assay
ERα: Estrogen receptor alpha
ERβ: Estrogen receptor beta
earlyFP: Early follicular phase
midFP: Mid follicular phase
lateFP: Late follicular phase
FP: Follicular phase
GC-MS/MS: Gas chromatography–tandem mass spectrometry
IUD: Intrauterine device
LC-MS/MS: Liquid chromatography–tandem mass spectrometry
LH: Luteinizing hormone
earlyLP: Early luteal phase
midLP: Mid luteal phase
lateLP: Late luteal phase
MC: Group: Eumenorrheic menstrual cycle
noP: No progesterone measurements
noT: No temperature measurements
OC: Oral contraceptive
OP: Ovulation phase
OSF: Open Science Framework
OSP: Olympic Training Center
SHBG: Sex hormone-binding globulin
