# Supplementary material for: Tracking androgens in female elite athletes: menstrual cycle and hormonal contraceptive effects
Source: BMC Womens Health. 2026 Mar 13;26:168. doi: 10.1186/s12905-026-04344-y (PMC13001183; doi:10.1186/s12905-026-04344-y)
Supplement: Supplementary file 1 — Supplementary Material 1. [file 12905_2026_4344_MOESM1_ESM.pdf]

Supplementary material

A1

Urine samples:

For the quantification of steroids in the urine samples, internal standards (testosterone-d3, epitestosterone-d3, androsterone-d4-glucuronide, 5β -androstanediol-d5, etiocholano-lon-d5) were added to 2 mL of urine. A pH value of 6.5 was achieved by adding phosphate buffer (0.5 M KH_2PO_4/ 0.5 M Na_2HPO_4, 8:5, v/v). This was followed by incubation with β-glucuronidase (from E. coli) at 50°C for 2 hours. After an adjustment to pH 9.0 by adding $NaHCO_3$/ K_2CO_3 (84:138, m/m), a liquid-liquid extraction was performed using 3 mL of methyl t-butyl ether. The organic phase was then evaporated under nitrogen. After derivatization with 40 µL MSTFA/NH_4I/propane-2-thiol 1000:5:1 v/m/v at 55°C for 30 min, analysis was performed by gas chromatography-coupled mass spectrometry (Triple Quad 7890A/7000 system, Agilent).

Table A2: *Descriptive urinary steroid concentrations across the 7-phase menstrual cycle model and pill phases. Values are presented as mean (MD) and standard deviation (Std.) for each group and phase. Concentrations are specific gravity–corrected and reported in ng/mL. Abbreviations: MC, eumenorrheic menstrual cycle; pill, combined oral contraceptive; free/use, pill-free vs pill-use phase; A, oligomenorrhea; C, short luteal phase; D, luteal phase deficiency; IUD1/IUD2, levonorgestrel IUD users (see Methods for details).*

	**Testosterone**		**5α - androstanedione**		**5β - androstanedione**		**5α -androstanediol**		**5β -androstanediol**		**Androsterone**		**Etiocholanolone**	
	**MD**	**Std.**	**MD**	**Std.**	**MD**	**Std.**	**MD**	**Std.**	**MD**	**Std.**	**MD**	**Std.**	**MD**	**Std.**
**MC**	**5,45**	**4,31**	**3,40**	**16,01**	**5,87**	**23,02**	**24,66**	**19,49**	**59,32**	**41,40**	**1997,14**	**1164,53**	**2246,59**	**1391,16**
earlyFP	4,64	4,05	6,05	16,10	10,98	27,45	23,30	19,70	58,26	51,47	1892,91	1225,10	2304,48	1639,73
midFP	5,52	3,80	1,68	3,92	2,80	2,34	26,41	17,53	59,48	38,11	2003,28	1112,90	2346,70	1480,48
lateFP	7,35	6,25	2,83	5,56	6,16	8,89	34,21	35,64	78,22	64,04	2393,48	1491,26	2691,69	1611,43
OP	6,38	4,61	10,94	43,59	16,15	54,53	26,25	20,02	56,27	30,11	2180,77	1240,13	2245,08	991,50
earlyLP	5,89	4,44	3,86	19,62	6,82	32,50	23,83	17,08	59,92	37,77	2030,81	1166,97	2145,96	1275,51
midLP	4,54	3,76	2,17	5,99	3,63	8,01	19,10	11,68	50,76	32,48	1835,93	879,89	1997,73	1177,49
lateLP	4,55	3,90	1,25	1,38	2,54	3,50	20,98	15,73	55,98	36,09	1823,20	1137,56	2044,22	1292,02
**pill**	**2,55**	**2,91**	**1,01**	**0,85**	**4,59**	**4,40**	**15,93**	**15,63**	**64,66**	**75,96**	**1804,04**	**1220,45**	**2260,78**	**1250,68**
free	2,56	2,95	1,02	1,01	4,00	4,25	16,05	16,13	68,68	82,80	1534,18	1092,72	2180,80	1272,84
use	2,55	2,91	1,01	0,81	4,75	4,43	15,90	15,54	63,53	74,18	1879,67	1246,67	2283,19	1247,60
**A**	**0,58**	**0,51**	**34,63**	**96,73**	**102,39**	**306,50**	**11,18**	**5,02**	**11,56**	**5,44**	**1573,23**	**627,24**	**964,13**	**402,50**
earlyFP	0,22	0,05	1,45	1,33	4,76	5,92	7,88	3,28	9,52	2,74	1241,18	401,94	783,80	247,75
midFP	0,59	0,49	38,74	103,51	115,86	328,11	11,45	5,16	11,53	5,56	1594,47	649,85	966,44	413,11
lateFP	1,11	0,87	18,72	19,22	33,33	24,50	11,79	4,74	15,14	7,32	1731,40	594,51	1197,69	460,61
OP														
earlyLP														
**C**	**4,91**	**2,77**	**1,17**	**1,15**	**2,55**	**2,24**	**19,83**	**13,25**	**56,21**	**39,35**	**1710,68**	**1038,87**	**2063,19**	**1153,08**
earlyFP	3,76	1,90	1,07	0,88	2,93	3,20	17,82	11,45	54,42	41,71	1508,63	741,83	1930,11	894,73
midFP	5,07	2,58	1,23	1,40	2,53	2,20	19,44	9,99	55,00	29,57	1658,40	998,98	2082,21	1072,85
lateFP	6,80	3,50	1,73	1,30	3,59	2,90	23,49	14,24	59,50	39,74	2207,49	1520,19	2546,42	1774,32

OP	4,53	3,66	0,92	0,63	1,78	1,37	15,35	11,32	39,69	27,61	1479,61	1163,82	1533,32	1018,90
earlyLP	4,99	2,63	0,92	0,63	2,19	1,21	22,02	17,25	59,25	45,48	1803,06	950,63	2075,24	1093,89
midLP	4,50	3,19	1,25	1,21	2,26	1,68	21,43	20,70	69,17	67,84	1732,25	1130,36	2093,70	1311,81
lateLP	4,20	1,53	0,98	1,22	2,48	2,73	15,07	6,64	52,37	20,16	1856,31	1261,16	2011,38	1423,08
**D**	**2,57**	**1,26**	**0,81**	**0,49**	**0,49**	**0,26**	**10,16**	**5,31**	**11,72**	**6,04**	**1021,23**	**486,84**	**366,08**	**188,90**
earlyFP	2,10	0,95	0,92	0,81	0,52	0,39	12,17	6,95	11,85	5,59	1176,10	681,16	398,07	227,47
midFP	2,49	1,00	0,94	0,47	0,45	0,20	9,30	4,14	10,52	4,98	888,02	338,63	321,04	135,68
lateFP	3,00	1,92	0,57	0,27	0,42	0,22	12,23	2,55	13,35	2,31	1207,17	904,88	434,80	296,16
OP	3,45	1,34	0,93	0,23	0,65	0,06	10,41	3,82	13,27	5,71	1414,60	581,33	574,67	263,34
earlyLP	2,23	1,15	0,62	0,31	0,42	0,12	10,77	8,42	14,31	10,12	987,95	505,24	352,24	204,66
midLP	2,62		0,76		0,83		8,14		10,46		838,98		323,23	
lateLP	2,93	1,95	0,70	0,54	0,53	0,40	9,05	5,06	10,55	6,04	1004,03	414,42	342,70	186,24
**IUD1**	**15,22**	**7,00**	**1,26**	**0,66**	**3,03**	**1,25**	**75,44**	**33,50**	**79,81**	**36,00**	**5233,38**	**2220,00**	**2266,11**	**1002,95**
AO	15,22	7,00	1,26	0,66	3,03	1,25	75,44	33,50	79,81	36,00	5233,38	2220,00	2266,11	1002,95
**IUD2**	**7,62**	**3,94**	**1,20**	**0,93**	**1,61**	**0,69**	**41,73**	**18,04**	**36,77**	**15,92**	**3829,34**	**1464,49**	**1744,35**	**759,77**
earlyFP	5,34	2,02	0,87	0,36	1,24	0,51	29,85	11,41	28,35	10,97	2838,57	1035,19	1427,16	595,15
midFP	7,37	3,38	1,27	1,34	1,65	0,62	43,83	21,22	35,26	16,11	3843,13	1752,23	1787,03	851,67
lateFP	12,16	2,22	2,15	0,87	2,88	0,76	61,63	8,43	63,39	11,85	5459,84	586,92	2752,54	566,72
OP	14,27	7,55	1,28	0,73	2,11	0,70	56,19	23,29	52,98	15,91	4236,53	2040,21	2006,17	1066,70
earlyLP	5,88	1,26	0,99	0,50	1,27	0,46	33,63	6,41	29,22	6,95	3418,05	672,42	1388,27	350,68
midLP	5,60	0,74	0,91	0,28	1,16	0,23	32,72	5,45	29,00	3,64	3393,97	344,07	1311,55	206,18
lateLP	8,37	3,00	1,25	0,61	1,73	0,23	49,53	20,01	43,88	15,75	4897,02	1738,52	2297,47	591,45

Table A3: *Overview of included data points and cycles per group and analyte. n_data indicates the number of included urine samples (daily measurements), and n_cycles indicates the number of menstrual/pill cycles contributing to the respective group.*

Group	Variable	n_data	n_cycles
A	Androsterone	37	1
A	Etiocholanolone	37	1
A	Testosterone	37	1
A	5α-androstanediol	37	1
A	5α-androstanedione	37	1
A	5β-androstanediol	37	1
A	5β-androstanedione	37	1
C	Androsterone	169	8
C	Etiocholanolone	169	8
C	Testosterone	169	8
C	5α-androstanediol	169	8
C	5α-androstanedione	169	8
C	5β-androstanediol	169	8
C	5β-androstanedione	169	8
D	Androsterone	43	2
D	Etiocholanolone	43	2
D	Testosterone	43	2
D	5α-androstanediol	43	2
D	5α-androstanedione	43	2
D	5β-androstanediol	43	2
D	5β-androstanedione	43	2
IUD1	Androsterone	42	1
IUD1	Etiocholanolone	42	1
IUD1	Testosterone	42	1
IUD1	5α-androstanediol	42	1
IUD1	5α-androstanedione	42	1
IUD1	5β-androstanediol	42	1
IUD1	5β-androstanedione	42	1
IUD2	Androsterone	52	2
IUD2	Etiocholanolone	52	2
IUD2	Testosterone	52	2
IUD2	5α-androstanediol	52	2
IUD2	5α-androstanedione	52	2
IUD2	5β-androstanediol	52	2
IUD2	5β-androstanedione	52	2
MC	Androsterone	332	15
MC	Etiocholanolone	332	15
MC	Testosterone	332	15
MC	5α-androstanediol	332	15
MC	5α-androstanedione	332	15
MC	5β-androstanediol	332	15
MC	5β-androstanedione	332	15
pill	Androsterone	201	10
pill	Etiocholanolone	201	10
pill	Testosterone	201	10
pill	5α-androstanediol	201	10
pill	5α-androstanedione	201	10
pill	5β-androstanediol	201	10
pill	5β-androstanedione	201	10

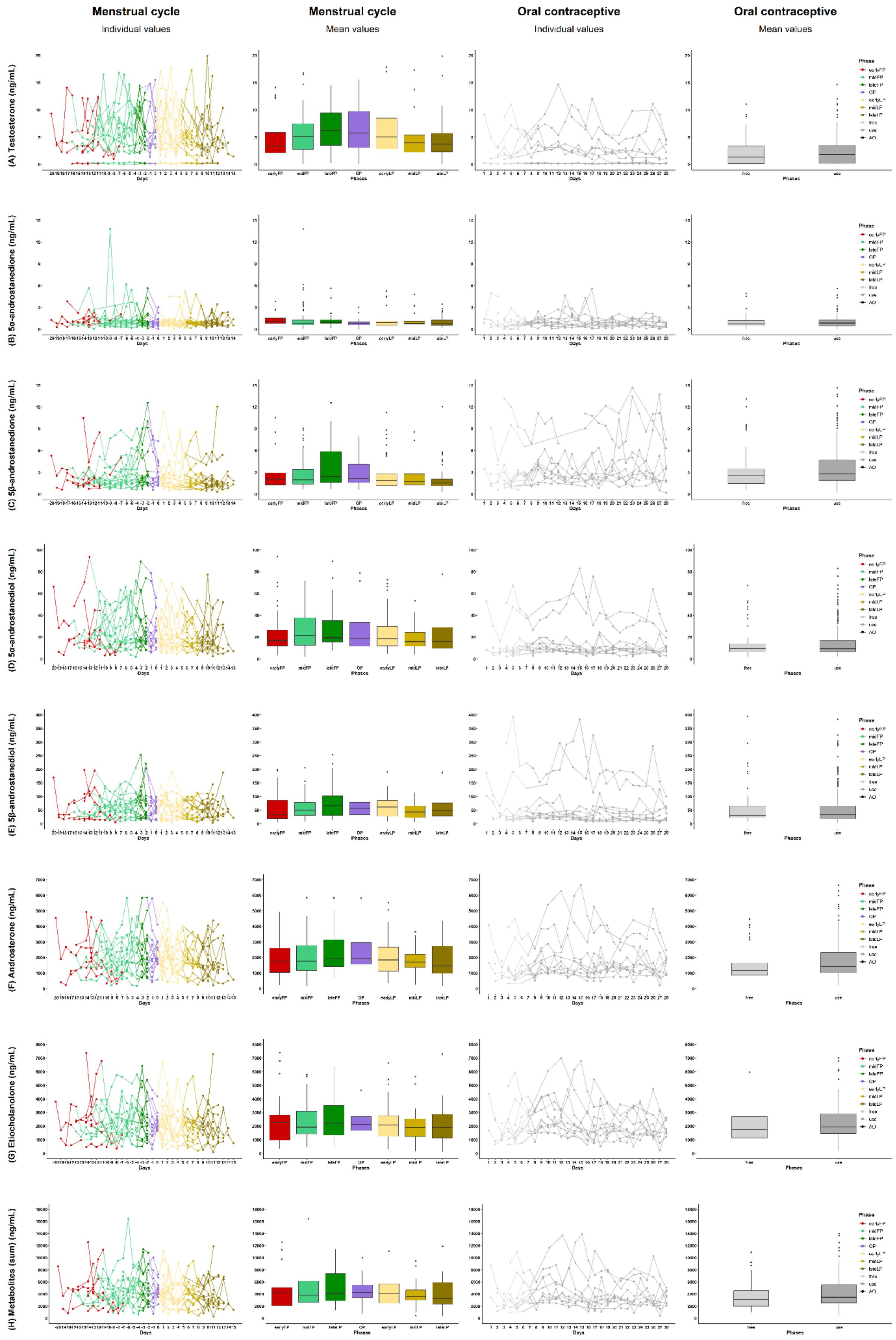

Figure A4: *Urinary androgen profile of menstrual cycle and oral contraceptive. The hormones presented*

include A: testosterone; B: 5α-androstanedione; C: 5β-androstanedione; D: 5α-androstanediol; E: 5β-androstanediol; F: androsterone; and G: etiocholanolone. Data is shown for two groups: individuals undergoing a natural menstrual cycle (colored lines and colored box plots, left) (ndata = 332; ncycle = 15) and individuals using combined oral contraceptives (grey lines and grey box plots, right) (ndata = 210; ncycle = 10) (see supplementary material A3). Hormone levels are plotted as line graphs across days normalised to the estimated day of ovulation (day: 0; FP < -1; LP > 1) and summarised as box plots across menstrual or pill phases. Axes are scaled per hormone to improve visualization of temporal patterns. Outliers were deleted only for visualization purposes. A lower-dose oral contraceptive (containing 0.1 mg levonorgestrel and 0.02 mg ethinylestradiol) is marked with triangles in the line plots.

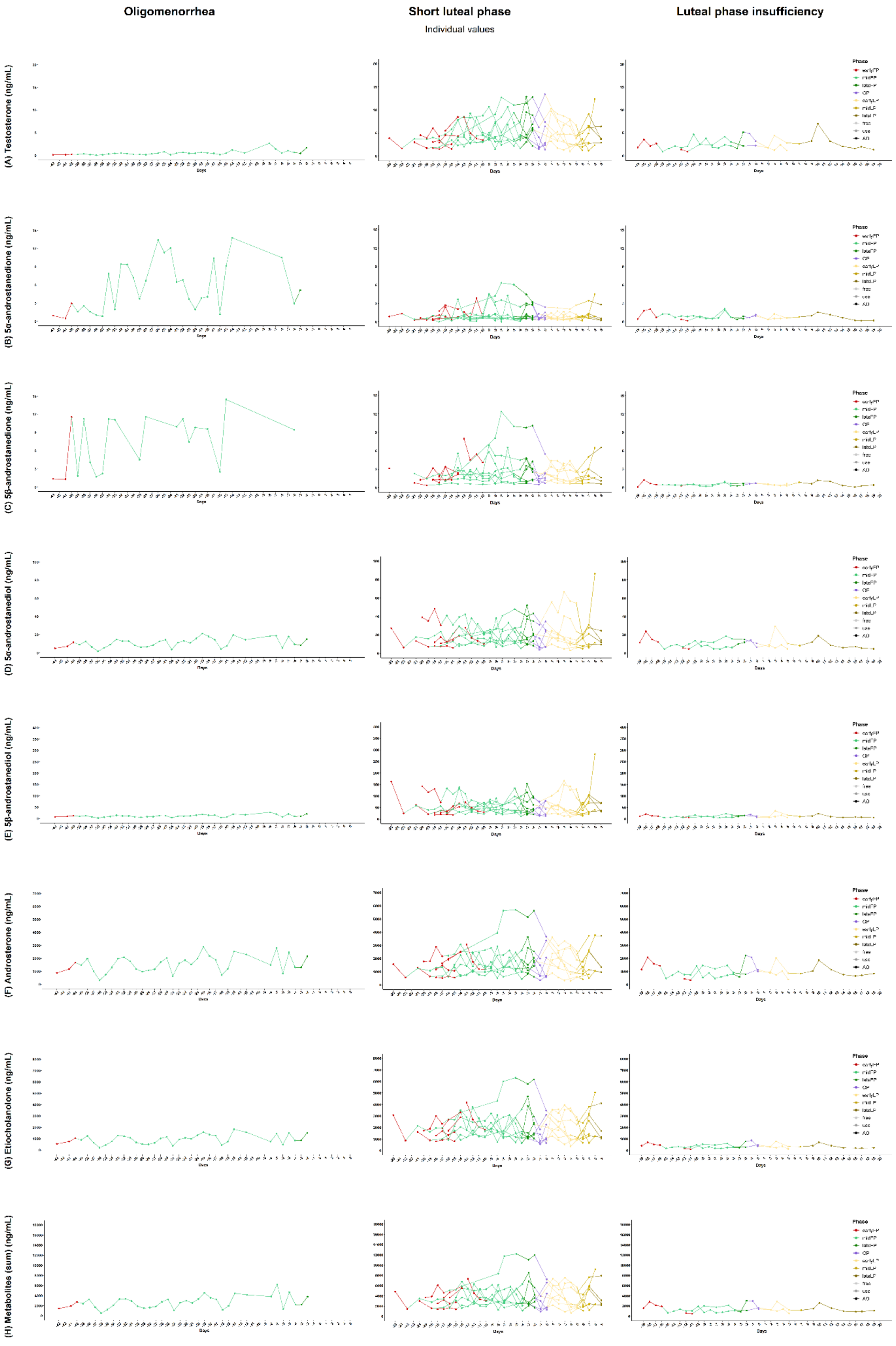

Figure A5: *Urinary androgen profiles across menstrual cycle disturbances. Data is grouped by cycle*

type: oligomenorrhea (left) (ndata = 37; ncycle = 1), short luteal phase (middle) (ndata = 169; ncycle = 8), and luteal phase insufficiency (right) (ndata = 43; ncycle = 2). The hormones presented include A: testosterone; B: 5α-androstanedione; C: 5β-androstanedione; D: 5α-androstanediol; E: 5β-androstanediol; F: androsterone; and G: etiocholanolone. Hormone concentrations are shown as line plots across days normalised to the estimated day of ovulation (day 0; FP < -1; LP > 1). Menstrual phases are color-coded in the plots. Axes are scaled per hormone to improve visualization of temporal patterns. Outliers were deleted only for visualization purposes.

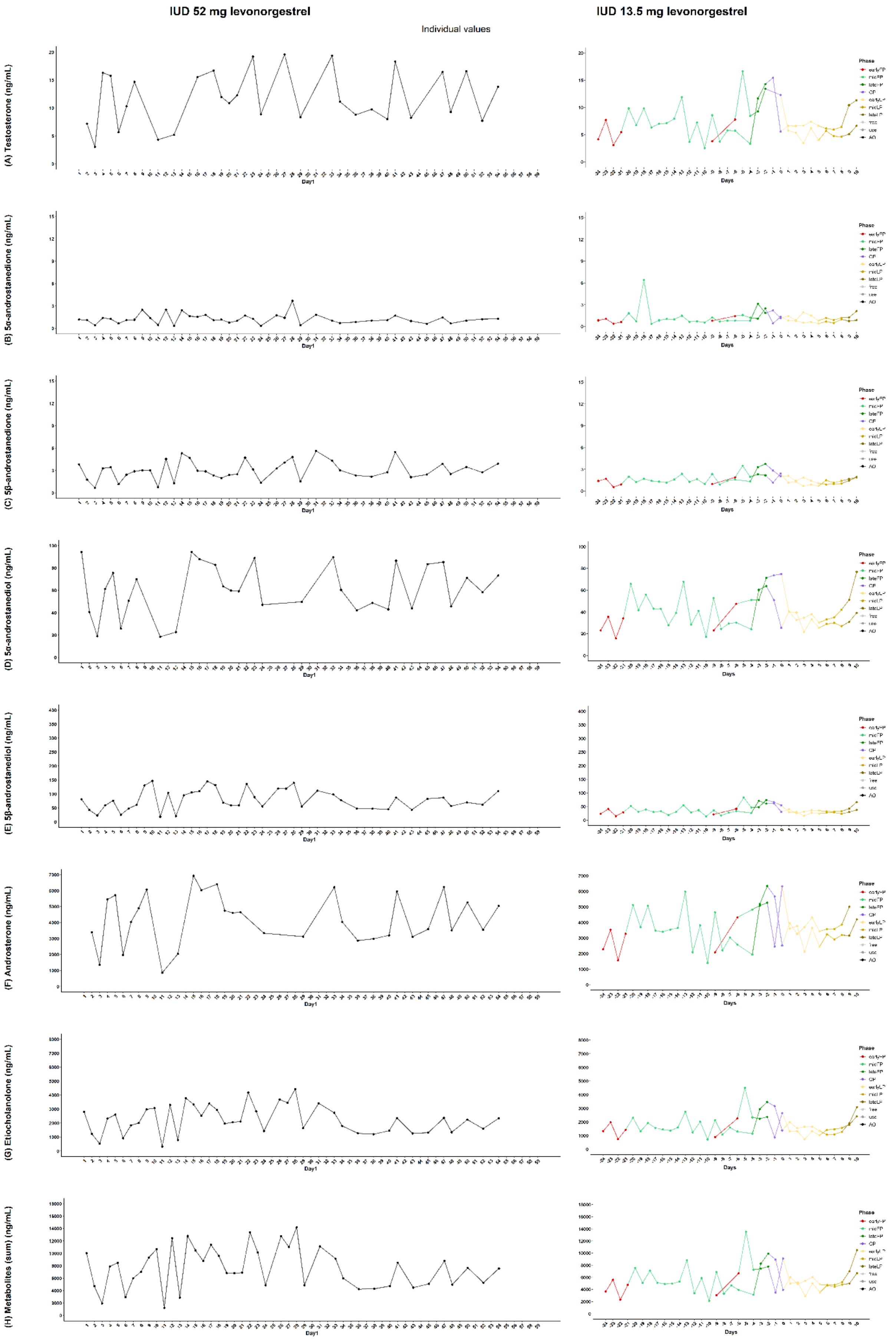

Figure A6: *Urinary androgen profiles in individuals using levonorgestrel-releasing intrauterine devices*

(IUDs). The hormones presented include A: testosterone; B: 5α-androstanedione; C: 5β-androstanedione; D: 5α-androstanediol; E: 5β-androstanediol; F: androsterone; and G: etiocholanolone. Line plots show hormone concentrations over time in users of either a high-dose IUD (52 mg levonorgestrel; left) (ndata = 42; ncycle = 1) or a lower-dose IUD (13.5 mg levonorgestrel; right) (ndata = 52; ncycle = 2). For the lower-dose IUD group, days are aligned to the estimated day of ovulation (day 0; FP < -1; LP > 1). In cases where ovulation did not occur, days are shown in chronological order. Axes are scaled per hormone to improve visualization of temporal patterns. Outliers were deleted only for visualization purposes.
